# Ecological host fitting of *Trypanosoma cruzi* TcI in Bolivia: mosaic population structure, hybridization and a role for humans in Andean parasite dispersal

**DOI:** 10.1111/mec.13186

**Published:** 2015-04-22

**Authors:** Louisa A. Messenger, Lineth Garcia, Mathieu Vanhove, Carlos Huaranca, Marinely Bustamante, Marycruz Torrico, Faustino Torrico, Michael A. Miles, Martin S. Llewellyn

**Affiliations:** ^1^ Department of Pathogen Molecular BiologyFaculty of Infectious and Tropical DiseasesLondon School of Hygiene and Tropical MedicineLondonUK; ^2^ Institute of Biomedical ResearchUniversidad Mayor de San SimónCochabambaBolivia; ^3^ Department of Infectious Disease EpidemiologyImperial College LondonLondonUK

**Keywords:** ecological fitting, microsatellites, mitochondria, population genetics, sylvatic transmission, *Trypanosoma cruzi*

## Abstract

An improved understanding of how a parasite species exploits its genetic repertoire to colonize novel hosts and environmental niches is crucial to establish the epidemiological risk associated with emergent pathogenic genotypes. *Trypanosoma cruzi*, a genetically heterogeneous, multi‐host zoonosis, provides an ideal system to examine the sylvatic diversification of parasitic protozoa. In Bolivia, *T. cruzi* I, the oldest and most widespread genetic lineage, is pervasive across a range of ecological clines. High‐resolution nuclear (26 loci) and mitochondrial (10 loci) genotyping of 199 contemporaneous sylvatic TcI clones was undertaken to provide insights into the biogeographical basis of *T. cruzi* evolution. Three distinct sylvatic parasite transmission cycles were identified: one highland population among terrestrial rodent and triatomine species, composed of genetically homogenous strains (*A*_r_ = 2.95; PA/L = 0.61; *D*_AS_ = 0.151), and two highly diverse, parasite assemblages circulating among predominantly arboreal mammals and vectors in the lowlands (*A*_r_ = 3.40 and 3.93; PA/L = 1.12 and 0.60; *D*_AS_ = 0.425 and 0.311, respectively). Very limited gene flow between neighbouring terrestrial highland and arboreal lowland areas (distance ~220 km; *F*_ST_ = 0.42 and 0.35) but strong connectivity between ecologically similar but geographically disparate terrestrial highland ecotopes (distance >465 km; *F*_ST_ = 0.016–0.084) strongly supports ecological host fitting as the predominant mechanism of parasite diversification. Dissimilar heterozygosity estimates (excess in highlands, deficit in lowlands) and mitochondrial introgression among lowland strains may indicate fundamental differences in mating strategies between populations. Finally, accelerated parasite dissemination between densely populated, highland areas, compared to uninhabited lowland foci, likely reflects passive, long‐range anthroponotic dispersal. The impact of humans on the risk of epizootic Chagas disease transmission in Bolivia is discussed.

## Introduction

Host–parasite relationships are assumed to be ecologically specialized, tightly co‐evolved systems driven by either mutual modification (synchronous cospeciation) or exaptation into novel environmental niches, often accompanied by host switching (ecological fitting) (Janzen 1985; Brooks *et al*. 2006). Ecological fitting occurs when an organism co‐opts an existing suite of genetic traits to exploit an unfamiliar resource or colonize and persist in a new or modified environment (Agosta & Klemens 2008). Distinguishing between host–parasite relationships that result from ecological fitting or long‐term co‐evolution remains challenging. However, understanding how a species exploits their current genetic repertoire to form novel host associations is of primary interest to the study of emerging infectious diseases, with considerable implications for the design of disease control programmes (Brooks & Ferrao 2005; Agosta *et al*. 2010). In this regard, *Trypanosoma cruzi* (Kinetoplastida: Trypanosomatidae), the aetiological agent of Chagas disease, a pervasive zoonosis that is eclectic with respect to host species and tissues it can inhabit, provides a model system to examine the genetic diversification of parasitic protozoa.

Chagas disease is the most important vector‐borne infection in Latin America, affecting an estimated 7–8 million individuals (World Health Organization 2014). Following acute disease, which is often undiagnosed, the majority of patients are clinically asymptomatic for life. Without treatment, approximately 20–30% will develop irreversible, potentially fatal cardiomyopathy or, more rarely, dilatation of the gastrointestinal tract (megaesophagus or megacolon) (Rassi *et al*. 2010). The geographical distribution of *T. cruzi* extends from the southern United States to Argentinean Patagonia, where it is transmitted by more than 100 species of hematophagus triatomine bugs (Hemiptera: Reduviidae: Triatominae) (Lent & Wygodzinsky 1979; Galvão *et al*. 2003). Human disease is primarily confined to areas of Central and South America where individuals are exposed to infected faeces of domiciliated or invasive triatomines through contact with intact mucosae or abraded skin (Coura & Dias 2009) or by oral ingestion of contaminated food/drink (Shikanai‐Yasuda & Carvalho 2012). In addition, enzootic *T. cruzi* infection is naturally sustained by an extensive range of domestic, synanthropic and sylvatic mammalian hosts (Noireau *et al*. 2009).

*Trypanosoma cruzi* is an ancient parasite, estimated to have diverged from its most recent common ancestor 3–4 Ma (Lewis *et al*. 2011), and as such, it is characterized by considerable genetic diversity (Stevens *et al*. 1999). Current international consensus recognizes a minimum of six stable genetic lineages or discrete typing units (DTUs) (TcI‐TcVI) (Zingales *et al*. 2009), which have distributions loosely defined by geography, ecology and transmission cycle (Miles *et al*. 2009). The level of nuclear sequence divergence between major *T. cruzi* DTUs is equivalent to interspecies diversity among New World *Leishmania* species (Yeo *et al*. 2011; Boité *et al*. 2012). TcI is the most widely distributed DTU; it is the principal cause of human chagasic cardiomyopathy in Colombia and Venezuela (Ramírez *et al*. 2010; Carrasco *et al*. 2012) and is ubiquitous among sylvatic transmission cycles across the parasite's endemic range (Llewellyn *et al*. 2009a). Multiple molecular markers consistently identify high levels of genetic diversity within sylvatic TcI populations (Herrera *et al*. 2007, 2009; O'Connor *et al*. 2007; Falla *et al*. 2009; Llewellyn *et al*. 2009a; Ocaña‐Mayorga *et al*. 2010; Lima *et al*. 2014), and divergent, but genetically homogeneous, strains isolated from human infections (Llewellyn *et al*. 2009a; Cura *et al*. 2010; Ramírez *et al*. 2012; Zumaya‐Estrada *et al*. 2012). However, the genetic determinants that drive natural *T. cruzi* diversification are largely unknown. Some have proposed that *T. cruzi* lineages co‐evolved in close concert with discrete vertebrate hosts and insect vectors (Miles *et al*. 1981; Gaunt & Miles 2000; Yeo *et al*. 2005), while others favour ecological fitting as a more parsimonious explanation for contemporary host associations (Hamilton *et al*. 2007; Agosta & Klemens 2008; Llewellyn *et al*. 2009a). Evidence to support the latter is increasing; TcI transmission is now known to span multiple ecological niches (Lisboa *et al*. 2004; Herrera *et al*. 2005, 2008a,b; Llewellyn *et al*. 2009a; Rocha *et al*. 2013; Lima *et al*. 2014), and genetic diversity of terrestrial TcIII appears similarly independent of host species (Llewellyn *et al*. 2009b; Marcili *et al*. 2009).

Bolivia comprises diverse sylvatic ecotopes where TcI transmission persists unabated and thus provides a perfect platform to test ecological hypotheses. Colonies of *Triatoma infestans*, infected with TcI (Barnabé *et al*. 2011; Breniere *et al*. 2012), have been reported in highland Andean valleys (Cortez *et al*. 2006, 2007; Buitrago *et al*. 2010) and to the south in the arid, lowland Chaco region (Ceballos *et al*. 2011; Waleckx *et al*. 2012), where their potential for domestic re‐invasion threatens the success of the National Control Programme (Noireau *et al*. 2005; Noireau 2009). Sylvatic TcI transmission also extends northwards to sparsely populated Amazonian Beni, where disease ecology is poorly described (Matias *et al*. 2003; Justi *et al*. 2010). Bolivia suffers the greatest human burden of *T. cruzi* infection in Latin America, impacting approximately 6.75% of the population (Jannin & Salvatella 2006). Chagas disease is endemic across two‐thirds of the country and concentrated disproportionally among lower socio‐economic rural populations with seroprevalence reaching 72.7–97.1% among adults of some communities (Medrano‐Mercado *et al*. 2008; Samuels *et al*. 2013). In these areas, continuing domestic transmission has been attributed to a decrease in intensity of residual insecticide spraying (Samuels *et al*. 2013; Espinoza *et al*. 2014), the emergence of insecticide resistance (Germano *et al*. 2010; Lardeux *et al*. 2010) and decentralized vector control initiatives in areas of recurrent political, social and economic instability (Gürtler 2009).

To date, few studies have adopted rigorous sampling strategies and genetic markers with sufficient resolution to elucidate fully the biogeographical basis of *T. cruzi* evolution. Ideally, parasite samples should be minimally subdivided biologically, spatially and temporally, with multiple clones examined from each host (Prugnolle & De Meeus 2010). In practice, low circulating parasitaemia often prohibits parasite isolation, and thus, many studies are heavily reliant on historical collections of reference isolates. *T. cruzi* genetic analysis is further complicated by the presence of mixed DTU infections (Bosseno *et al*. 1996; Yeo *et al*. 2005; Burgos *et al*. 2008) and multiclonal parasite populations within individual hosts and vectors (Llewellyn *et al*. 2011), requiring strains to be biologically cloned prior to genotyping, a laborious caveat often overlooked by researchers.

In this study, we applied high‐resolution nuclear and mitochondrial genotyping to contemporaneous biologically cloned TcI strains, isolated from triatomines and mammalian hosts across Bolivia, to identify key determinants of sylvatic *T. cruzi* genetic diversification. We also explore genetic diversity and potential hybridization along two ecological clines, first between highland and lowland ecotopes and second within lowland Bolivia itself. Finally, we examine the spatial genetic structure of natural TcI populations and consider the implications of our data for human Chagas disease transmission in Bolivia.

## Materials and methods

### Study area and parasite sampling

**Figure 1:**
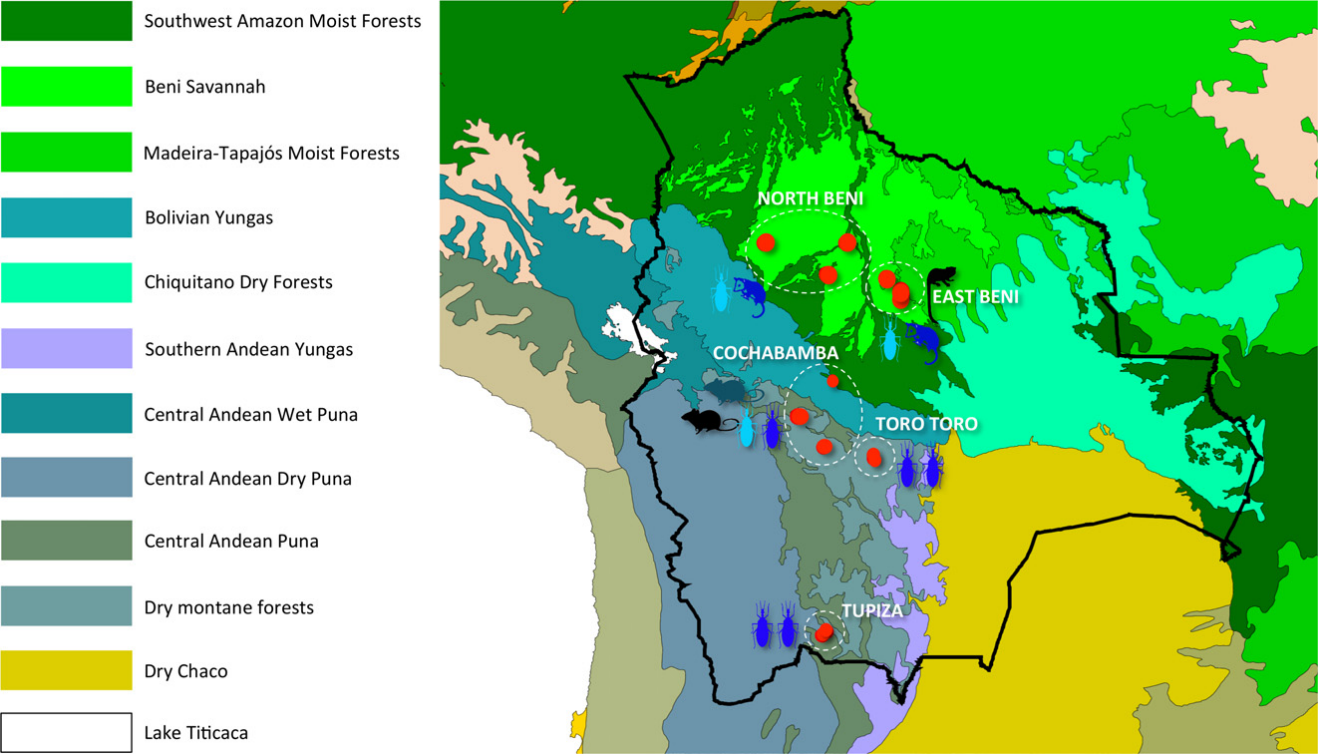
Map of Bolivia showing distribution of sylvatic TcI isolates among different ecotopes. Parasite strains were isolated from terrestrial and arboreal transmission cycles in five localities across three departments: Cochabamba, Potosí and Beni. Study sites were situated at altitudes that ranged from ~143 to 3200 m and spanned five different ecoregions: savannah grassland and Madeira‐Tapajós moist forests (Beni), dry Andean puna and Yungas (Cochabamba) and dry montane forests (Potosí). Geographical origins of individual strains are shown by closed red circles. Circle areas are proportionate to sampling density. Images indicate sample host/vector origin (rodent, marsupial, primate or triatomine). Open white circles designate five a priori populations: Cochabamba, Tupiza, Toro Toro, North Beni and East Beni used for population genetic analyses. Population and department names are indicated in uppercase and lowercase, respectively.

Parasite strains were isolated from sylvatic terrestrial and arboreal transmission cycles in five localities across three departments in Bolivia (Cochabamba, Potosí and Beni) (Fig. 1). Study sites were situated at altitudes that ranged from ~143 to 3200 m and selected to span five major ecoregions: savannah grassland and Madeira‐Tapajós moist forests (Beni), dry Andean puna and Yungas (Cochabamba) and wet Andean puna (Potosí). Triatomine vectors were sampled using a combination of manual microhabitat dissection and live‐baited Noireau traps (Noireau *et al*. 2002). Wild mammalian reservoir hosts were captured using Sherman and Tomahawk box and cage traps. Parasite sampling was undertaken from 2004 to 2010 and is described for each study site individually.

The Cotopachi site in Cochabamba department is located in a densely populated area of open dry Andean puna (thorny scrub vegetation interspersed with rocky outcrops and large, spiny cacti), ~20 km southwest of Cochabamba city at an elevation of ~2600 m. Here, TcI parasites were sampled from wild *Triatoma infestans* and terrestrial rodents (*Akodon boliviensis* and *Phyllotis osilae*). Triatomine sampling (*T. infestans* and *T. guasayana*) was also undertaken in neighbouring Toro Toro, an area of similar ecology to Cotopachi, situated at ~2700 m. North of Cotopachi, sylvatic *Rhodnius robustus* were collected from Chapare, a dense temperate montane forest (Yungas) in the westernmost foothills of the Andes.

South of Cochabamba, TcI parasites were isolated from wild *T. infestans* in Tupiza, a region of high‐altitude (~3200 m) montane grasslands in Potosí department.

Sampling was undertaken in two regions of Beni department, a sparsely populated province in eastern lowland Bolivia. Ecologically, Beni is a patchwork of two principal vegetation types. The majority of the department is covered by lush savannah grassland (Llanos de Moxos). Along riverine alluvial plains and to the northern and western borders of the area, this ecotope is supplanted by dense Amazonian moist forests. To the east, Beni borders another moist forest (Madeira‐Tapajós), which extends into Brazil and Santa Cruz department. In East Beni (Nueva Alianza, San Juan de Aguas Dulces and San Juan de Mocovi), TcI parasites were isolated from triatomines (*Rhodnius pictipes*) and mammals (*Didelphis marsupialis*,* Philander opossum* and *Sciureus* species) in areas of savannah grassland, interspersed with large stands of evergreen palm trees, on the boundary between Llanos de Moxos and the moist forests of northwestern Santa Cruz. The study sites in North Beni (Mercedes, San Cristobal and Santa Maria de Apere) were remote, largely uninhabited, open savannah grasslands with occasional lone standing trees, bordered by riverine forests. Here TcI parasites were isolated from *R. robustus*,* P. opossum* and *D. marsupialis*. Both study sites in North and East Beni were situated at low‐lying altitudes (~143 and ~160 m, respectively), and parasite sampling was undertaken using similar methods described for other departments.

All parasite strains were isolated by direct inoculation of triatomine faeces or heparinized venous animal blood into biphasic hemoculture media (Miles 1993).

### Parasite strains and DTU confirmation

A panel of 199 biological clones derived from 68 *Trypanosoma cruzi* TcI isolates was assembled for analysis (Table S1, Supporting information). Biological clones were obtained from primary cultures by plate cloning according to Yeo *et al*. (2007) to minimize any loss of genetic diversity incurred by long‐term maintenance in culture. Parasites (epimastigotes) were expanded to logarithmic phase at 28 °C in RPMI‐1640 liquid media supplemented with 0.5% (w/v) tryptone, 20 mm HEPES buffer (pH 7.2), 30 mm haemin, 10% (v/v) heat‐inactivated foetal calf serum, 2 mm sodium glutamate, 2 mm sodium pyruvate and 25 μg/mL gentamycin (all Sigma‐Aldrich, UK). Genomic DNA was extracted using the Gentra PureGene Tissue Kit (Qiagen, UK), according to the manufacturer's protocol. Clone genotypes were confirmed as TcI using a triple‐marker assay (Lewis *et al*. 2009), to exclude the potential presence of mixed infections with several DTUs, and classified a priori into five populations according to geographical origin: Cochabamba (*n* = 28), Tupiza (*n* = 15), Toro Toro (*n* = 43), North Beni (*n* = 26) and East Beni (*n* = 87).

### High‐resolution genotyping: multilocus microsatellite typing

Twenty‐six microsatellite loci were amplified for all 199 clones, as previously described by Llewellyn *et al*. (2009a). These markers were distributed across 10 putative chromosomes, including six groups of physically linked loci (Weatherly *et al*. 2009). A full list of microsatellite targets and primers are given in Table S2 (Supporting information). Allele sizes were determined using an automated capillary sequencer (AB3730, Applied Biosystems, UK), in conjunction with a fluorescently tagged size standard, and were manually checked for errors. All isolates were typed ‘blind’ to control for user bias.

### High‐resolution genotyping: mitochondrial multilocus sequence typing

Ten maxicircle gene fragments were sequenced for a subset of 78 clones, chosen to be representative of total nuclear genetic diversity (indicated in Table S1, Supporting information) (Messenger *et al*. 2012). For NADH dehydrogenase subunit 4 (*ND4*), an alternate set of primers was designed to improve amplification efficiency: *ND4* forward (5′‐TTYTTCCCAATATGTATBGTMAG‐3′) and *ND4* reverse (5′‐TGTATTAYCGAYCAATTYGC‐3′), and reactions were performed using the same conditions as previously (Messenger *et al*. 2012).

### Microsatellite analysis

Individual‐level sample clustering was initially defined using a neighbour‐joining (NJ) tree based on pairwise distances (*D*_AS_: 1 − proportion of shared alleles at all loci/*n*) between microsatellite genotypes calculated in microsat v1.5d (Minch *et al*. 1997) under the infinite‐alleles model. To accommodate multi‐allelic genotypes (≥3 alleles per locus), a script was written in Microsoft Visual Basic to generate random multiple diploid resamplings of each multilocus profile (software available upon request). A final pairwise distance matrix was derived from the mean across multiple resampled data sets and used to construct a NJ phylogenetic tree in phylip v3.67 (Felsenstein 1989). Majority rule consensus analysis of 10 000 bootstrap trees was performed in phylip v3.67 by combining 100 bootstraps generated in microsat v1.5d, each drawn from 100 respective randomly resampled data sets.

A second analysis to define the number of putative populations in the data set was performed using a nonparametric approach (free from Hardy–Weinberg assumptions). A *K*‐means clustering algorithm, implemented in adegenet (Jombart 2008), was used to determine the optimal number of ‘true’ populations, with reference to the Bayesian information criterion (BIC), which reaches a minimum when approaching the best supported assignment of individuals to the appropriate number of clusters. The relationship between these clusters and the individuals within them was then evaluated via a discriminant analysis of principal components (DAPC) according to Jombart *et al*. (2010).

A single randomly sampled diploid data set was used for all subsequent analyses (Appendix S1, Supporting information; available from Dryad doi: 10.5061/dryad.b8465). Population‐level genetic diversity was evaluated using sample‐size‐corrected allelic richness (*A*_r_) in fstat 2.9.3.2 (Goudet 1995). In addition, mean *F*_IS_, which measures the distribution of heterozygosity within and between individuals, was calculated per population in fstat 2.9.3.2. *F*_IS_ can vary between −1 (all loci are heterozygous for the same alleles) and +1 (all loci are homozygous for different alleles). *F*_IS_ = 0 indicates Hardy–Weinberg allele frequencies. Sample‐size‐corrected private (population‐specific) allele frequency per locus (PA/L) was calculated in hp‐rare (Kalinowski 2005).

Population subdivision was estimated using pairwise *F*_ST_, linearized with Slatkin's correction, in arlequin v3.11 (Excoffier *et al*. 2005). Statistical significance was assessed via 10 000 random permutations of alleles between populations. Within‐population subdivision was evaluated in arlequin v3.11 using a hierarchal analysis of molecular variance (amova). Population‐level heterozygosity indices were also calculated in arlequin v3.11 and associated significance levels for *P*‐values derived after performing a sequential Bonferroni correction to minimize the likelihood of type I errors (Rice 1989). Multilocus linkage disequilibrium, estimated by the index of association (*I*_A_), was calculated in multilocus 1.3b (Agapow & Burt 2001), and statistical significance was evaluated by comparison with a null distribution of 1000 randomizations. Mantel's tests for the effect of isolation by distance (IBD) within populations (pairwise genetic vs. geographical distance) were implemented in genaiex 6.5 using 10 000 random permutations (Peakall & Smouse 2012).

### Mitochondrial analysis

Sequence data from 10 maxicircle gene fragments were concatenated for each isolate according to Messenger *et al*. (2012) and are available from Dryad (doi: 10.5061/dryad.b8465). Additional mitochondrial multilocus sequence typing (mtMLST) data from 24 previously published TcI strains were included in selected analyses, as indicated (Messenger *et al*. 2012). The most appropriate nucleotide substitution model was selected from 1624 candidates, based on the Akaike information criterion (AIC), in jmodeltest 2.1.4 (Darriba *et al*. 2012). Alternate maximum‐likelihood (ML) phylogenies were constructed using the TrN+G model (six substitution rate categories) in mega 5.10 (Tamura *et al*. 2011). Bootstrap support for clade topologies was estimated following the generation of 1000 pseudoreplicate data sets. Bayesian phylogenetic analysis was performed with mrbayes, implemented through topali v2.5, using the best‐fit model based on the BIC (GTR+G) (Milne *et al*. 2009). Five independent analyses were run for one million generations, with sampling every 100 simulations (30% burn‐in). Statistically supported topological incongruence between alternate mitochondrial and nuclear phylogenies was evaluated using Kishino–Hasegawa (KH) (Kishino & Hasegawa 1989) and Shimodaira–Hasegawa (SH) (Shimodaira & Hasegawa 1999) likelihood tests in paml v.4 (Yang 2007). Haplotype diversity (*H*_d_) was calculated using dnasp v5.10.1 (Librado & Rozas 2009).

## Results

### Strain characteristics

One hundred and ninety‐nine biological clones were genotyped across 26 polymorphic microsatellite loci (Appendix S1, Supporting information). In total, 10 122 alleles were identified, corresponding to 178 unique multilocus genotypes (MLGs). Multiple (≥3) alleles were observed at 0.83% of loci. Levels of intrastrain genetic diversity were high; multiclonality, that is the presence of multiple, different genetic clones, was observed in 65/68 strains. Identical intraclonal genotypes were sampled in five isolates (1/18 Toro Toro, 1/11 Cochabamba and 3/26 East Beni). Clones were initially categorized into five populations based on geographical origin, consisting of three high‐altitude (Cochabamba, Tupiza and Toro Toro) and two low‐altitude groups (North and East Beni). All populations demonstrated uniformly high numbers of unique MLGs and low frequencies of repeated MLGs (Table 1).

**Table 1:** Population genetic parameters for sylvatic populations of *Trypanosoma cruzi* TcI in Bolivia

Population^a^	G/N	Max. Freq. MLG	*H*_d_ (H/N)	PL	PA/L ±SE	*A*_r_ ±SE	*H*_o_	*H*_e_	% *H*_e_	% *H*_d_	*F*_IS_ ±SE	*I*_A_	*I*_A_ *P‐*value
All highlands	75/86	3	0.54 (9/46)	21	0.61 ± 0.15	2.95 ± 0.37	0.26	0.23	33.3	19	−0.158 ± 0.02	2.06	<0.001
Cochabamba (highlands)	25/28	2	0.40 (4/14)	20	0.42 ± 0.12	2.22 ± 0.20	0.29	0.24	30	5	−0.206 ± 0.10	2.56	<0.001
Tupiza (highlands)	14/15	2	0.73 (3/6)	15	0.21 ± 0.07	2.21 ± 0.29	0.28	0.28	6.7	13.3	0.026 ± 0.08	3.54	<0.001
Toro Toro (highlands)	39/43	2	0.46 (4/26)	18	0.19 ± 0.06	1.92 ± 0.21	0.25	0.20	22.2	11.1	−0.241 ± 0.09	1.48	<0.001
North Beni (lowlands)	22/26	2	0.81 (4/7)	19	0.60 ± 0.16	3.93 ± 0.39	0.37	0.45	10.5	63.2	0.176 ± 0.06	2.70	<0.001
East Beni (lowlands)	78/87	3	0.84 (9/25)	21	1.12 ± 0.29	3.40 ± 0.46	0.39	0.48	9.5	52.3	0.203 ± 0.05	2.23	<0.001

*N*, number of isolates in population; G, number of multilocus genotypes (MLGs) per population based on microsatellite data of 26 loci analysed; Max. Freq. of MLG, frequency of the most common MLG within the population; *H*, number of haplotypes in population; *H*_d_, haplotype diversity measures the uniqueness of a particular haplotype in a given population, calculated using available mitochondrial sequence data in dnasp v5.10.1 (Librado & Rozas 2009); PL, number of polymorphic loci out of 26 loci analysed; *A*_r_, allelic richness as a mean over loci ±SE, calculated in fstat 2.9.3.2 (Goudet 1995); PA/L, mean number of private alleles per locus ±SE, calculated in HP‐Rare (Kalinowski 2005); *H*_o_, mean observed heterozygosity across all loci; *H*_e_, mean expected heterozygosity across all loci; %*H*_E_, proportion of loci showing a significant excess in heterozygosity after a sequential Bonferroni correction (Rice 1989); %*H*_d_, proportion of loci showing a significant deficit in heterozygosity after a sequential Bonferroni correction (Rice 1989); *F*_IS_, mean fixation index ±SE, calculated in fstat 2.9.3.2 (Goudet 1995); *I*_A_, index of association calculated in multilocus 1.3b; *P*‐value estimated by comparison with a null distribution of 1000 randomizations (Agapow & Burt 2001); DAPC, discriminant analysis of principal components.

a Population designation based on a priori geographical populations and DAPC/*D*_AS_ strain assignments.

### Nuclear genetic clustering among isolates

**Figure 2:**
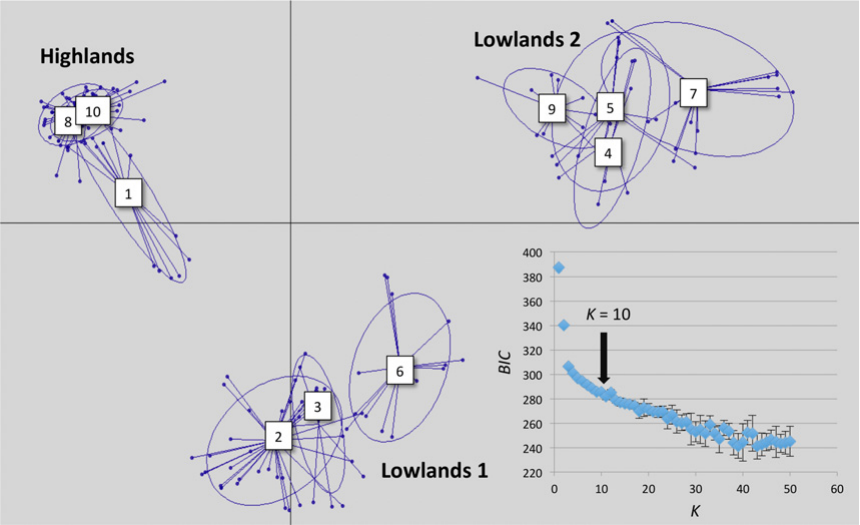
Nuclear genetic clustering among 199 sylvatic Bolivian TcI clones. Multidimensional scaling plot based on discriminant analysis of principal component (DAPC) analysis for 10 clusters defined via *K*‐means clustering algorithm (10^9^ iterations, three principal components representing 80% of total variation in the data set). Bayesian information criterion (BIC) curve is inserted with error bars representing the standard deviation about the mean of five independent runs. Inertia ellipses correspond to the optimal (as defined by the BIC minimum) number of population clusters among the genotypes analysed. Individual clones are indicated by dots. The 10 DAPC clusters are separated into three genetically distinct groups: highlands (clusters 1, 8 and 10), lowlands 1 (clusters 2, 3 and 6) and lowlands 2 (clusters 4, 5, 7 and 9).

Patterns of isolate clustering were evaluated using two different methodologies: nonparametric population assignment (DAPC) and a NJ analysis based on pairwise genetic distances (*D*_AS_). Ten genetic clusters were defined among the 199 clones submitted to DAPC, once three principal components (PCs) were retained and analysed (representing 80% of the total variation). A full list of isolate assignments to DAPC populations is included in Table S1 (Supporting information), and a multidimensional scaling plot of the DAPC results is shown in Fig. 2. We observed a slight ‘elbow’ in the distribution of the BIC values across optimal cluster numbers at *K *=* *10 (Fig. 2). DAPC‐derived clusters were largely congruent with a priori allocations of strains to geographical populations. The 10 DAPC clusters separated into three genetically distinct groups: highlands (clusters 1, 8 and 10), lowlands 1 (clusters 2, 3 and 6) and lowlands 2 (clusters 4, 5, 7 and 9). The highlands group corresponded exclusively to samples from Cochabamba, Tupiza and Toro Toro, with the exception of a single clone from *Rhodnius robustus* in Chapare (CV‐05 cl1), which was instead assigned to cluster 2 in the lowlands 1 group. Within the highlands group, isolates from different sampling areas and sources (hosts and vectors) were distributed across clusters 8 and 10, while cluster 1 comprised only a subset of clones from *Triatoma infestans* found in Tupiza and Toro Toro. The lowlands 1 group encompassed all strains from North Beni (only cluster 2) and approximately half of the isolates from *Rhodnius* spp. and *Didelphis marsupialis* in East Beni (interspersed among clusters 2, 3 and 6). Lastly, the lowlands 2 group contained all remaining East Beni clones, including those isolated from *Rhodnius* spp., *D. marsupialis*,* Philander opossum* and *Sciureus* spp.

**Figure 3:**
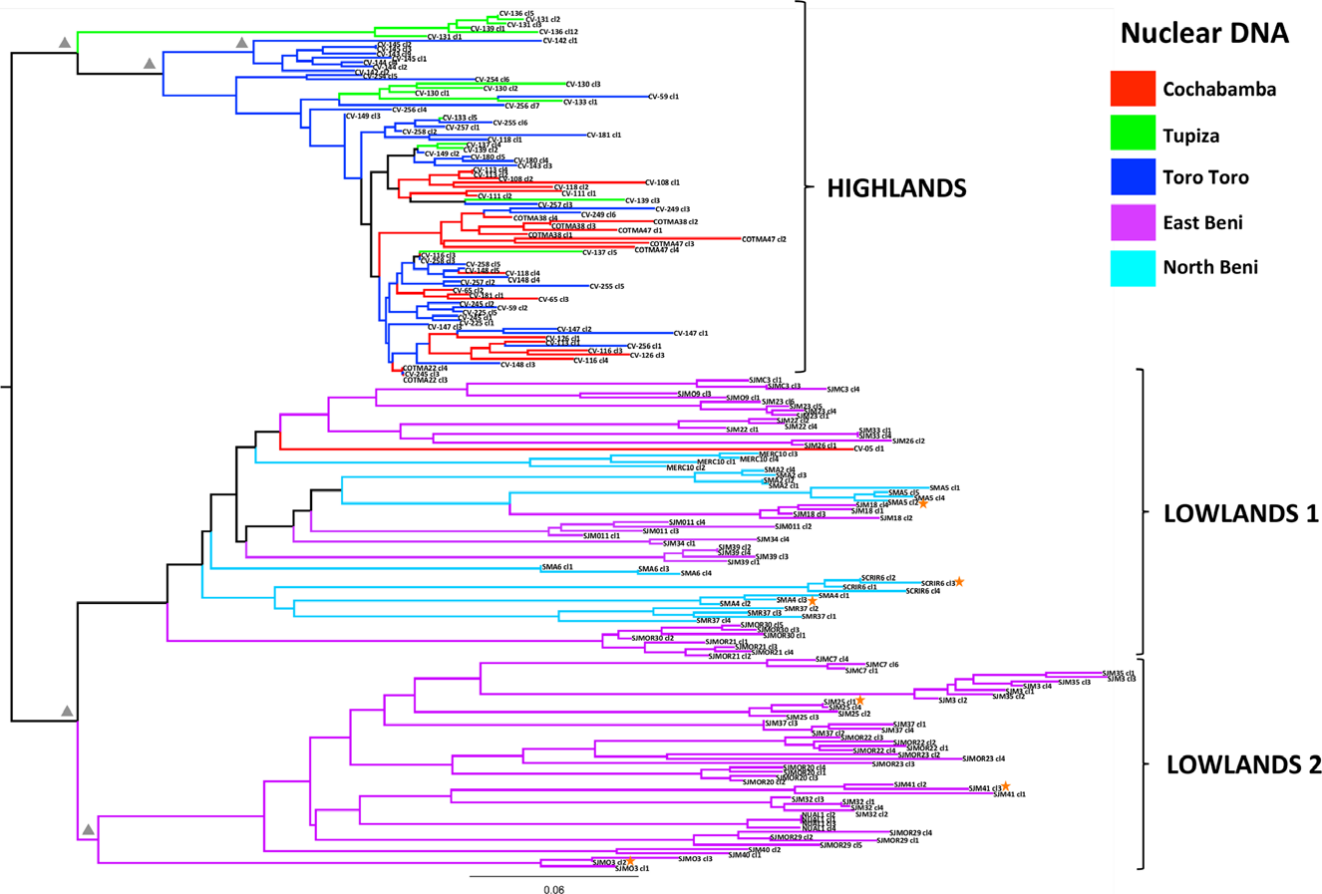
Unrooted neighbour‐joining tree based on *D*_AS_ values between multilocus genotypes generated for 199 sylvatic Bolivian TcI clones. *D*_AS_ values were calculated as the mean across 1000 random diploid resamplings of the data set. Branch colours indicate isolate a priori population (Cochabamba, Tupiza, Toro Toro, East Beni and North Beni; see legend). Closed grey triangles are adjacent to nodes that receive >60% bootstrap support. Isolates are grouped into three statistically supported clades (highlands, lowlands 1 and lowlands 2). Orange stars denote clones which have phylogenetically incongruent positions between nuclear and mitochondrial topologies.

A NJ tree based on the same microsatellite data was constructed and further corroborated the DAPC strain assignments. A clear division between highland and lowland populations was observed, with isolates segregating into two well‐supported clades (64% BS) (Fig. 3). Similar to the DAPC results, the *D*_AS_ topology supported the delineation of isolates from Beni into two groups (71% BS), one composed of all North Beni clones and the same portion of East Beni clones (*D*_AS_ lowlands 1), the other containing the remaining East Beni strains (*D*_AS_ lowlands 2). As previously, CV‐05 cl1 from Chapare clustered as an outlier among North and East Beni isolates. Comparison of branch lengths in Fig. 3 between the two lowland populations indicated high and consistent levels of genetic variation across strains. By contrast, highland isolates were less diverse overall (mean pairwise *D*_AS_ = 0.151 and 0.431 for highlands and lowlands, respectively). Within this clade, there was strong evidence for the existence of local geographical clusters in Tupiza (100% BS) and Toro Toro (73% BS), which clustered basally to the remaining highland strains.

### Population characteristics

**Figure 4:**
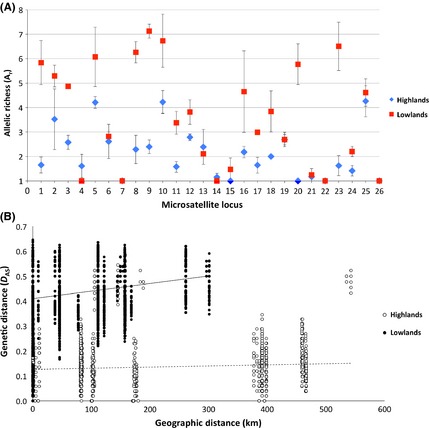
(A) Allelic richness (*A*_r_) per microsatellite locus for grouped a priori geographical highland (diamonds) and lowland (squares) populations. Highland populations were characterized by smaller estimates of allelic richness (*A*_r_), compared to the lowlands (average of *A*_r_ = 1.92–2.22 and 3.40 and 3.93, respectively). Error bars represent ±SE about the mean. Values without error bars correspond to markers containing only a single variable locus. (B) Nuclear spatial genetic analysis among *Trypanosoma cruzi* isolates from highland (open circles) and lowland (closed circles) populations. Nuclear genetic isolation by distance (IBD) was observed among lowland populations (*R*_XY_ = 0.209, *P *<* *0.001; slope = 0.0003 ± 0.0000179), while no spatial structure was evident among highland populations spanning a much greater geographical area (*R*_XY_ = 0.109, *P *=* *0.085; slope = 0.0002 ± 0.0000307).

Population genetic indices were calculated using both a priori geographical and DAPC/*D*_AS_‐supported strain assignments (Table 1). Overall, a clear division in genetic diversity and heterozygosity was apparent between highland and lowland areas. The three highland populations were characterized by lower levels of genetic diversity, as evidenced by smaller estimates of allelic richness (*A*_r_ = 1.92–2.22) and numbers of private alleles per locus (PA/L = 0.19–0.42), compared to the lowlands (*A*_r_ = 3.40 and 3.93 and PA/L = 1.12 and 0.60, respectively) (Table 1 and Fig. 4A). All highland groups had moderately excess heterozygosity (*F*_IS_ = −0.241 to 0.026, 5–13.3% polymorphic loci with significant deficit in heterozygosity), whereas both lowland populations demonstrated more pronounced deviations from H–W allele frequencies (*F*_IS_ = 0.176 and 0.203, 63.2% and 52.3% polymorphic loci with significant deficit in heterozygosity, respectively) (Table 1). Strongly significant multilocus linkage disequilibrium was observed among all study areas (*I*_A_ = *P *<* *0.0001 for all populations).

### Interpopulation gene flow and intrapopulation subdivision

Estimates of subdivision (*F*_ST_) between a priori populations support a genetic demarcation between highland and lowland areas (Table 2). Little evidence for subdivision existed among the three highland study sites (*F*_ST_ = 0.084, 0.016 and 0.079 and *P *=* *0.00089, 0.0032 and 0.0001 for Cochabamba–Tupiza, Cochabamba–Toro Toro and Tupiza–Toro Toro, respectively) or between the two lowland populations (*F*_ST_ = 0.087 and *P *<* *0.0001 for North and East Beni). However, elevated *F*_ST_ values between closest highland and lowland study sites (Cochabamba–Beni distance = ~220 km; *F*_ST_ = 0.42 and 0.35 and *P *<* *0.0001 for Cochabamba–North and East Beni, respectively) indicate very limited gene flow, suggesting a powerful role for altitude and/or ecotope in structuring parasite populations. Interestingly, the extent of genetic subdivision between the most geographically distant highland populations (Cochabamba–Tupiza; distance = ~465 km) and adjacent areas of Beni (distance = ~155 km) was equivalent (*F*_ST_ = 0.084 and 0.087, respectively).

**Table 2:** *F*_ST_ values in a five way comparison between populations (*P*‐value indicated in brackets)

	Cochabamba (highlands)	Tupiza (highlands)	Toro Toro (highlands)	North Beni (lowlands)	East Beni (lowlands)
Cochabamba (highlands)	*				
Tupiza (highlands)	0.084 (0.00089 ± 0.0003)	*			
Toro Toro (highlands)	0.016 (0.00317 ± 0.0006)	0.079 (0.00010 ± 0.0001)	*		
North Beni (lowlands)	0.42 (0.000 ± 0.000)	0.25 (0.000 ± 0.000)	0.50 (0.000 ± 0.000)	*	
East Beni (lowlands)	0.35 (0.000 ± 0.000)	0.26 (0.000 ± 0.000)	0.40 (0.000 ± 0.000)	0.087 (0.000 ± 0.000)	*

Finally, a hierarchical amova was conducted, to evaluate the distribution of genetic diversity between groups of populations (highlands vs. lowlands), among populations within groups (Cochabamba, Tupiza, Toro Toro, North Beni and East Beni) and among individuals within populations. Strikingly, 23% of total genetic variation was attributed to differences between highlands and lowlands, while 4.5% and 7% were present at the population and the individuals within population levels, respectively.

### Mitochondrial introgression across ecological clines

**Figure 5:**
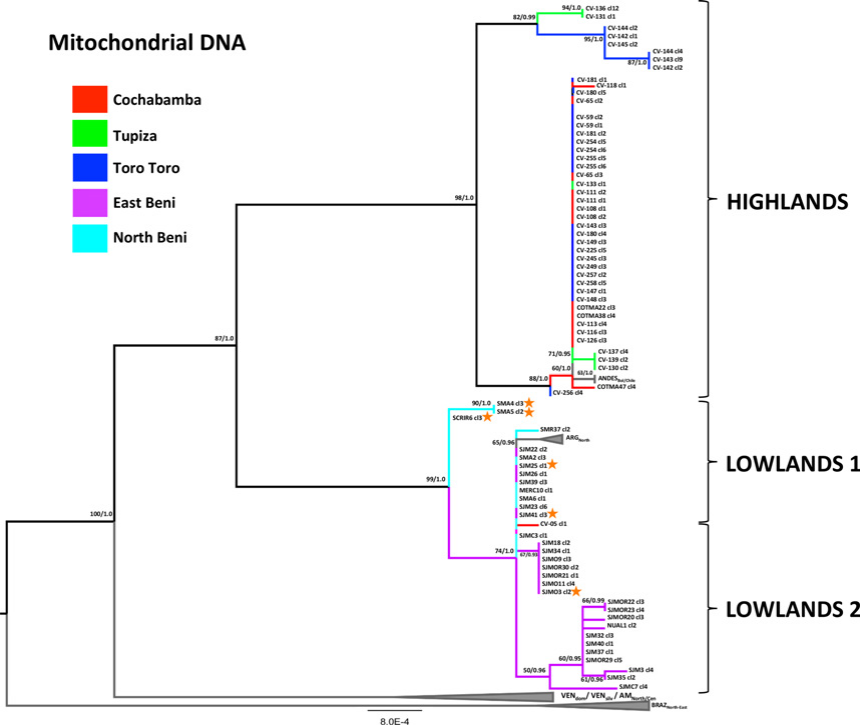
Maximum‐likelihood (ML) tree constructed from concatenated maxicircle sequences for 78 sylvatic Bolivian TcI clones and 24 additional TcI isolates from across the Americas. A ML topology was constructed from concatenated maxicircle sequences for 78 sylvatic Bolivian TcI clones and rooted using 24 additional TcI strains belonging to six previously characterized populations (AM_N__orth/Cen_, ANDES_B__ol/Chile_, ARG_N__orth_, BRAZ_N__orth‐East,_ VEN_dom_ and VEN_silv_ from Messenger *et al*. 2012). The most appropriate nucleotide substitution model was TrN+G (six substitution rate categories) based on the Akaike information criterion. Branch colours indicate sample a priori population (Cochabamba, Tupiza, Toro Toro, East Beni and North Beni; see legend). Statistical support for major clades are given as equivalent bootstraps and posterior probabilities from consensus ML (1000 pseudoreplicates) and Bayesian trees (based on the GTR+G model), respectively. Orange stars denote clones which have statistically supported phylogenetically incongruent positions between nuclear and mitochondrial topologies.

For a subset of 78 clones, 10 mitochondrial gene fragments (mtMLST) were sequenced and concatenated into a 3684‐bp alignment. Twenty‐four unique haplotypes were identified from a total of 48 variable sites (~1.3% sequence diversity). ML and Bayesian phylogenies constructed from concatenated data were not significantly different (KH test: ML tree *L *=* *−4845.23, Bayesian tree *L *=* *−4848.13, *P *=* *0.12). A second ML tree was assembled using 24 additional outgroup sequences representing known TcI mitochondrial diversity, including a small population of domestic Bolivian isolates (ANDES_Bol/Chile_, previously described in Llewellyn *et al*. 2009a and Messenger *et al*. 2012) (Fig. 5).

The mitochondrial topology demonstrated the presence of considerable genetic variation among Bolivian TcI clones. The deepest and most robust internal branch (87/1.0) separated highland and lowland populations into two major clades, each with strongly supported internal structuring. The highland group was largely homogeneous, with a number of geographically dispersed strains sharing identical mitochondrial haplotypes. The mitochondrial topology also confirmed the existence of Tupiza‐ and Toro Toro‐specific populations (98/1.0), in agreement with the nuclear tree.

Human isolates from Cochabamba (ANDES_Bol/Chile_), while genetically distinct from sylvatic strains circulating in the same area (63/1.0), were grouped within the main highlands clade. As previously, lowland strains were subdivided into two well‐supported clades (74/1.0) with higher overall levels of genetic diversity, compared to highland isolates (*H*_d_ = 0.81 and 0.84 vs. 0.54, respectively; Table 1).

While the gross topology of the mitochondrial tree was broadly concordant with that of the nuclear phylogeny, internal branch patterns were significantly incongruent (SH test: ML tree *L *=* *−4845.86, Bayesian tree *L *=* *−4849.55 and *D*_AS_ tree *L *=* *−5006.48, *P *=* *0.001). No evidence of recombination between highland and lowland strains was observed, even in Chapare, a zone of ecological transition. Across the more ‘gentle’ ecological cline of East–North Beni, several instances of genetic hybridization were apparent. Three clones from the mixed East–North Beni group (*D*_AS_ lowlands 1) and three isolates from the East Beni‐specific population (*D*_AS_ lowlands 2) received unambiguously different phylogenetic positions in the maxicircle topology and are likely the progeny of multiple, independent mitochondrial introgression events (Fig. 5).

### Geographical dispersal within populations

To determine the extent of spatial genetic structure (or IBD) among highland and lowland isolates, Mantel's tests were conducted using alternate nuclear and mitochondrial data sets. Nuclear IBD was detected within both highland and lowland populations (highland *R*_XY_ = 0.307, *P *<* *0.001, and lowland *R*_XY_ = 0.209, *P *<* *0.001). However, the strength of the effect was significantly larger among lowland isolates (highland slope = 0.0002 ± 0.00000873; lowland slope = 0.0003 ± 0.0000179). Furthermore, when focusing on highland clones from approximately the same spatial scale as their lowlands counterparts [i.e. omitting the local subpopulation of Tupiza isolates identified in the *D*_AS_ tree (*n* = 6)], little evidence for spatial structuring remained (*R*_XY_ = 0.109, *P *=* *0.085). Concordant with estimates of *F*_ST_ between populations, the differing extent of spatial genetic structuring suggests accelerated parasite dispersal among geographically disparate highland areas by comparison with adjacent lowland foci (Fig. 4B).

Interestingly, no IBD was detected in either highland (*R*_XY_ = 0.068, *P *=* *0.161; slope = 0.000001 ± 0.000000345) or lowland (*R*_XY_ = 0.119, *P *=* *0.0654; slope = 0.000001 ± 0.000000349) populations using mitochondrial sequence data, potentially the result of lower population genetic resolution at these loci, but also consistent with the occurrence of mitochondrial introgression among lowland isolates.

## Discussion

This study exploited rigorous population genetic analyses of contemporaneous parasite clones. Herein, we provide several insights into the biogeographical basis of *Trypanosoma cruzi* genetic diversification in Bolivia. Additionally, our study undertook an in‐depth dissection of TcI spatial genetic diversity and hybridization across two ecological clines.

### Lowland arboreal and highland terrestrial sylvatic populations show different genetic structures

A clear dichotomy in population structure emerged between highland and lowland areas. Lowland parasites from two adjacent arboreal transmission cycles were strongly subdivided within a restricted contact zone in East Beni (~15 km^2^). Deep internal nuclear branching patterns in both lowland groups were indicative of stable, undisturbed, long‐term genetic diversification, with correspondingly high levels of diversity. Mitochondrial introgression occurring among genetically distinct strains in Beni supports prolonged historical interactions between these two populations. Consistent with high intrahost and vector clonal diversity, these data support intense, local transmission and/or low rate of genotypic extinction (Criscione & Blouin 2006). MLGs were rarely repeated, indicating only a fraction of total population genetic diversity was sampled.

In contrast, highland populations were considerably less diverse compared to their lowland counterparts and widespread dispersal of genetically homogeneous strains was observed across geographically disparate terrestrial highland populations, supported by little evidence of genetic substructuring (low *F*_ST_). Dissimilar heterozygosity estimates between highlands (excess) and lowlands (deficit) suggest a recent hybrid origin for some highland strains or fundamental differences in mating systems between these two populations (Ramírez & Llewellyn 2014). Importantly, human isolates from Cochabamba were closely related to adjacent sylvatic highland strains.

Gross differences between highland and lowland population structures may be partially explained in the context of their respective ecological niches. Most lowland parasites were isolated from *Didelphimorphia* mammals, prominent disease reservoirs which are susceptible to high circulating parasitaemia (Legey *et al*. 2003) and have a propensity for nonvectoral routes of infection, including oral transmission via predation of infected vectors or mammals (Jansen & Roque 2010; Rocha *et al*. 2013) and exposure to contaminated anal scent gland secretions (Carreira *et al*. 2001). These biological features may predispose these hosts to multiplicity of infection which will be directly related to intensity and efficiency of parasite transmission and duration and course of disease (Roellig *et al*. 2010; Nouvellet *et al*. 2013). The high levels of genetic diversity among Bolivian lowland strains are consistent with this hypothesis.

While minimal parasite interaction was observed between neighbouring terrestrial and arboreal transmission cycles (high *F*_ST_ values between Cochabamba and Beni), a single clone (CV‐05 cl1) isolated from *Rhodnius robustus* in the Andean foothills was more closely related to lowland Beni strains on the basis of both nuclear and mitochondrial markers, suggesting the existence of an additional, under‐sampled transmission cycle and potential hybridization zone in Chapare, northern Cochabamba.

The remaining lowland strains were isolated from *Rhodnius* vectors (*R. robustus* and *Rhodnius pictipes*). In general, sylvatic *Rhodnius* species are promiscuous feeders, which can actively migrate at night to colonize domestic environments (Feliciangeli *et al*. 2007; Fitzpatrick *et al*. 2008), thus promoting the accumulation of mixed DTU infections (Bosseno *et al*. 1996; Yeo *et al*. 2005), as well as infrahost multiclonality and co‐infections with other trypanosome species, such as *Trypanosoma rangeli* (Dias *et al*. 2014). The lower genetic diversity observed among highlands strains may reflect more restricted feeding preferences and limited independent dispersal of their host vector species *Triatoma infestans* (<500 m) (Rabinovich & Himschoot 1990; Richer *et al*. 2007). As a more recent host of TcI, vector competency of sylvatic *T. infestans* may also vary, particularly in terms of bottlenecks during transmission, which can further reduce genetic diversity, as demonstrated in tsetse fly vectors of other digenetic trypanosome species (Ruepp *et al*. 1997; Oberle *et al*. 2010).

### Ecological fitting is a driver of contemporary *T. cruzi* genetic diversification

No clear association of genotype by host or vector was observed among any sylvatic Bolivian TcI population, with the exception of a small subset of coclustering *T. infestans* clones sampled in Tupiza and Toro Toro (DAPC cluster 1 and *D*_AS_ highlands; *n* = 14). Previous *T. cruzi* studies that favoured constrained, extant co‐evolutionary scenarios were probably limited by sampling bias (O'Connor *et al*. 2007); *Didelphimorphia* mammals continue to be oversampled as sources of sylvatic TcI due to their aforementioned high circulating parasitaemia, which can facilitate greater hemoculture positivity rates and thus parasite isolation, as well as their ease of capture.

With improved and more exhaustive sampling strategies, TcI has now been detected among a range of *Mammalia* (Lisboa *et al*. 2004; Herrera *et al*. 2005, 2008a,b; Yeo *et al*. 2005; Rocha *et al*. 2013; Lima *et al*. 2014), cautioning the interpretation of putative host associations. Here we demonstrate that parasite genetic diversity was principally partitioned by ecotope: arboreal lowland or terrestrial highland. Limited gene flow between neighbouring arboreal and terrestrial transmission cycles and low levels of subdivision among similar ecotopes, spanning much larger geographical distances (*F*_ST_), strongly suggest ecological host fitting is the predominant mechanism of sylvatic *T. cruzi* diversification (Llewellyn *et al*. 2009a,b). Our observations support a current model for wider trypanosome evolution where ecological host fitting has been proposed to define major parasite clades (Hamilton *et al*. 2007; Lukes *et al*. 2014).

### Mitochondrial introgression is a common phenomenon among natural *T. cruzi* populations

The majority of field evidence indicates *T. cruzi* does not conform to strict clonality or panmixia and that recombination is common, nonobligatory and idiosyncratic, potentially involving independent exchange of kinetoplastid and nuclear genetic material and both canonical meiotic and parasexual mechanisms (Carrasco *et al*. 1996; Machado & Ayala 2001; Ocaña‐Mayorga *et al*. 2010; Lewis *et al*. 2011; Messenger *et al*. 2012; Ramírez *et al*. 2012; Roellig *et al*. 2013; Baptista Rde *et al*. 2014). The relative contributions of alternate mating strategies to *T. cruzi* population structures are as yet unclear and strongly debated (Tibayrenc & Ayala 2012, 2013; Ramírez & Llewellyn 2014).

One aim of our study was to evaluate the extent of genetic recombination within two putative hybrid zones. Due to limited sample size (only a single isolate could be recovered from the politically unstable Chapare region), we were unable to detect hybridization across the highland–lowland cline. However, mitochondrial introgression was observed among a subset of lowland strains between East and North Beni. Evidence of intra‐TcI genetic exchange in a primary Amazonian forest (Carrasco *et al*. 1996), between domestic/peri‐domestic populations in Ecuador (Ocaña‐Mayorga *et al*. 2010) and within an endemic focus in Colombia (Ramírez *et al*. 2012) suggests that intensive local sampling of transmission cycles is an effective strategy to detect recombination.

Arboreal lowland populations in Beni provide an example of an undisturbed epidemiological situation where genetic exchange might be expected (Carrasco *et al*. 1996). Two divergent TcI populations overlap in this region, one sharing affinities to TcI populations from the Chaco region to the South (East Beni), the other more related to Amazonian TcI to the North (North Beni) (Llewellyn *et al*. 2009a; Lima *et al*. 2014). Experimental recombination in *T. cruzi* was shown to arise in mammalian cell cultures (Gaunt *et al*. 2003). The aforementioned *Didelphimorphia* maintain high levels of multiclonal parasite populations, providing ample opportunities for hybridization to occur. Multiple mitochondrial introgression events were detected in East Beni, which appeared independent of parasite nuclear genotype, mammalian host species and study site. Consistent with previous studies, no evidence of reciprocal nuclear hybridization was detected among recombinant strains (Messenger *et al*. 2012; Ramírez *et al*. 2012). While the biological cues that initiate genetic exchange remain unresolved (Gaunt *et al*. 2003; Lewis *et al*. 2010), in these populations we speculate that asymmetric introgression may act as a mechanism to facilitate ecological fitting (e.g. host range extension or resource tracking), considering the crucial role that mitochondria play in parasite metabolism, growth and development and their elevated need to escape Muller's ratchet compared to the nuclear genome (Neiman & Taylor 2009; Ramírez & Llewellyn 2014).

### Dispersal of Chagas disease in highland Bolivia

Multiple lines of evidence suggest that there is no ‘bona fide’ sylvatic transmission cycle in the Bolivian highlands. Little spatial differentiation was detected among geographically disparate highland populations (~465 km), and this level was comparable to that observed between neighbouring lowland areas (~155 km). Terrestrial clones also displayed limited genetic IBD, a lack of private alleles and excess heterozygosity, all potentially attributable to a recent population bottleneck and/or founder event followed by clonal propagation.

The putative accelerated parasite dispersal between highland sites in comparison with lowland areas does not accord with the ecology expected for local established sylvatic transmission. Indeed, didelphid marsupials and *Rhodnius* vectors have a far greater capacity for auto‐dissemination than *T. infestans* and smaller rodents (Richer *et al*. 2007). Instead, parasite dispersal across the highlands may be recent and anthroponotic. Substantial population genetic evidence indicates that *T. infestans* has a precedent for passive dissemination by human populations throughout history, initially during pre‐Incan times throughout the Western Andes (Schofield 1988; Bargues *et al*. 2006; Cortez *et al*. 2010) and subsequently, post‐Colombian, eastwards into Argentina, Paraguay, Uruguay and Brazil (Panzera *et al*. 2004; Piccinali *et al*. 2009). Trafficking of genetically homogeneous, human‐infective (at least in Cochabamba), highland TcI clones is reminiscent of the epidemic propagation of hybrid *T. cruzi* lineages TcV and TcVI by domestic *T. infestans* across the Southern Cone (Lewis *et al*. 2011). All highland study sites coincided with major, densely populated, transport routes transecting the departments of Cochabamba and Potosí and the distribution of highland strains closely reflected human migratory movements.

Genetic continuity between human and sylvatic strains in the highlands adjacent to Cochabamba by mitochondrial MLST confirms the existence of gene flow from local sylvatic to domestic transmission cycles. More widespread highland domestic infestation with *T. infestans* might be expected if its sylvatic distribution was the result of anthropogenic propagation. Thus, the extent to which humans are responsible for long‐range parasite distribution throughout highland Bolivia remains to be resolved. Importantly, the widespread dispersal of limited diversity genotypes in Bolivia has significant biological and medical implications with respect to virulence, transmissibility and drug susceptibility, and the potential risk for emergent epizootic Chagas disease.

L.A.M. designed and performed the experiments, analysed the data and drafted the manuscript. L.G. participated in fieldwork, contributed materials and analysed the data. M.V. contributed materials and analysed the data. C.H., M.B. and M.T. participated in fieldwork. F.T. contributed materials. M.A.M. drafted the manuscript. M.S.L. designed the study, participated in fieldwork, analysed the data and drafted the manuscript.

## Data accessibility

Strain panel (Table S1, Supporting information), microsatellite primers (Table S2, Supporting information), microsatellite genotypes (Appendix S1, Supporting information), concatenated maxicircle sequence alignment, mitochondrial ML phylogeny and microsatellite NJ phylogeny are all available from Dryad: doi: 10.5061/dryad.b8465.

## Supporting information


**Appendix S1** Dataset

**Table S1** Panel of Bolivian *T. cruzi* TcI biological clones assembled for analysis.

**Table S2** Panel of microsatellite loci and primers employed in this study.
