# Supplementary material for: Ecological host fitting of Trypanosoma cruzi TcI in Bolivia: mosaic population structure, hybridization and a role for humans in Andean parasite dispersal
Source: Mol Ecol. 2015 Apr 22;24(10):2406–22. doi: 10.1111/mec.13186 (PMC4737126; doi:10.1111/mec.13186)
Supplement: Supplementary file 2 — Table S1 Panel of Bolivian T. cruzi TcI biological clones assembled for analysis. [file MEC-24-2406-s002.docx]

**Table S1.** Panel of Bolivian *T. cruzi* TcI biological clones assembled for analysis.

| Strain Code | Date of Isolation | Host/Vector | Geographical Origin | Latitude^a^ | Longitude^a^ | *a priori* Population^b^ | DAPC Cluster^c^ | *D*_AS_ Population^d^ |
| --- | --- | --- | --- | --- | --- | --- | --- | --- |
| COTMA22 cl3^*^ | 11.10.04 | *Akodon boliviensis* | Cotopachi, Cochambamba | -17.43 | -66.283 | Cochabamba | 8 | Highlands |
| COTMA22 cl4 | 11.10.04 | *Akodon boliviensis* | Cotopachi, Cochambamba | -17.43 | -66.283 | Cochabamba | 8 | Highlands |
| COTMA38 cl1 | 13.10.04 | *Akodon boliviensis* | Cotopachi, Cochambamba | -17.43 | -66.283 | Cochabamba | 8 | Highlands |
| COTMA38 cl2 | 13.10.04 | *Akodon boliviensis* | Cotopachi, Cochambamba | -17.43 | -66.283 | Cochabamba | 8 | Highlands |
| COTMA38 cl3 | 13.10.04 | *Akodon boliviensis* | Cotopachi, Cochambamba | -17.43 | -66.283 | Cochabamba | 8 | Highlands |
| COTMA38 cl4^*^ | 13.10.04 | *Akodon boliviensis* | Cotopachi, Cochambamba | -17.43 | -66.283 | Cochabamba | 10 | Highlands |
| COTMA47 cl1 | 13.10.04 | *Phyllotis ocilae* | Cotopachi, Cochambamba | -17.43 | -66.283 | Cochabamba | 8 | Highlands |
| COTMA47 cl2 | 13.10.04 | *Phyllotis ocilae* | Cotopachi, Cochambamba | -17.43 | -66.283 | Cochabamba | 10 | Highlands |
| COTMA47 cl3 | 13.10.04 | *Phyllotis ocilae* | Cotopachi, Cochambamba | -17.43 | -66.283 | Cochabamba | 10 | Highlands |
| COTMA47 cl4^*^ | 13.10.04 | *Phyllotis ocilae* | Cotopachi, Cochambamba | -17.43 | -66.283 | Cochabamba | 8 | Highlands |
| CV-05(172) cl1^*^ | 3.06.09 | *Rhodnius robustus* | Chapare/San Julian | -16.7167 | -65.6333 | Cochabamba | 2 | Lowlands 1 |
| CV-065(278) cl2^*^ | 11.02.10 | *Triatoma infestans* | Quillacolla/Cotapachi | -17.4249 | -66.2947 | Cochabamba | 8 | Highlands |
| CV-065(278) cl3^*^ | 11.02.10 | *Triatoma infestans* | Quillacolla/Cotapachi | -17.4249 | -66.2947 | Cochabamba | 10 | Highlands |
| CV-108(270) cl1^*^ | 10.03.10 | *Triatoma infestans* | Quillacolla/Cotapachi | -17.4249 | -66.2947 | Cochabamba | 8 | Highlands |
| CV-108(270) cl2^*^ | 10.03.10 | *Triatoma infestans* | Quillacolla/Cotapachi | -17.4249 | -66.2947 | Cochabamba | 8 | Highlands |
| CV-111(271) cl1^*^ | 10.03.10 | *Triatoma infestans* | Quillacolla/Cotapachi | -17.4249 | -66.2947 | Cochabamba | 10 | Highlands |
| CV-111(271) cl2^*^ | 10.03.10 | *Triatoma infestans* | Quillacolla/Cotapachi | -17.4249 | -66.2947 | Cochabamba | 10 | Highlands |
| CV-113(298) cl1 | 10.03.10 | *Triatoma infestans* | Quillacolla/Cotapachi | -17.4249 | -66.2947 | Cochabamba | 8 | Highlands |
| CV-113(298) cl2 | 10.03.10 | *Triatoma infestans* | Quillacolla/Cotapachi | -17.4249 | -66.2947 | Cochabamba | 8 | Highlands |
| CV-113(298) cl4^*^ | 10.03.10 | *Triatoma infestans* | Quillacolla/Cotapachi | -17.4249 | -66.2947 | Cochabamba | 8 | Highlands |
| CV-116(251) cl3^*^ | 10.03.10 | *Triatoma infestans* | Quillacolla/Cotapachi | -17.4249 | -66.2947 | Cochabamba | 8 | Highlands |
| CV-116(251) cl4 | 10.03.10 | *Triatoma infestans* | Quillacolla/Cotapachi | -17.4249 | -66.2947 | Cochabamba | 10 | Highlands |
| CV-116(251) cl5 | 10.03.10 | *Triatoma infestans* | Quillacolla/Cotapachi | -17.4249 | -66.2947 | Cochabamba | 10 | Highlands |
| CV-118(272) cl1^*^ | 10.03.10 | *Triatoma infestans* | Quillacolla/Cotapachi | -17.424 | -66.2934 | Cochabamba | 10 | Highlands |
| CV-118(272) cl2 | 10.03.10 | *Triatoma infestans* | Quillacolla/Cotapachi | -17.424 | -66.2934 | Cochabamba | 8 | Highlands |
| CV-118(272) cl4 | 10.03.10 | *Triatoma infestans* | Quillacolla/Cotapachi | -17.424 | -66.2934 | Cochabamba | 10 | Highlands |
| CV-126(303) cl1 | 11.03.10 | *Triatoma infestans* | Quillacolla/Cotapachi | -17.424 | -66.2934 | Cochabamba | 8 | Highlands |
| CV-126(303) cl3^*^ | 11.03.10 | *Triatoma infestans* | Quillacolla/Cotapachi | -17.424 | Quillacolla | Cochabamba | 8 | Highlands |
| CV-130(256) cl1 | 1.04.10 | *Triatoma infestans* | Tupiza/Urulica | -21.5944 | -65.8322 | Tupiza | 10 | Highlands |
| CV-130(256) cl2^*^ | 1.04.10 | *Triatoma infestans* | Tupiza/Urulica | -21.5944 | -65.8322 | Tupiza | 10 | Highlands |
| CV-130(256) cl3 | 1.04.10 | *Triatoma infestans* | Tupiza/Urulica | -21.5944 | -65.8322 | Tupiza | 10 | Highlands |
| CV-131(252) cl1^*^ | 1.04.10 | *Triatoma infestans* | Tupiza/Urulica | -21.5944 | -65.8322 | Tupiza | 1 | Highlands |
| CV-131(252) cl2 | 1.04.10 | *Triatoma infestans* | Tupiza/Urulica | -21.5944 | -65.8322 | Tupiza | 1 | Highlands |
| CV-131(252) cl3 | 1.04.10 | *Triatoma infestans* | Tupiza/Urulica | -21.5944 | -65.8322 | Tupiza | 1 | Highlands |
| CV-133(254) cl1^*^ | 1.04.10 | *Triatoma infestans* | Tupiza/Urulica | -21.5944 | -65.8322 | Tupiza | 10 | Highlands |
| CV-133(254) cl5 | 1.04.10 | *Triatoma infestans* | Tupiza/Urulica | -21.5944 | -65.8322 | Tupiza | 8 | Highlands |
| CV-136(258) cl12^*^ | 1.04.10 | *Triatoma infestans* | Tupiza/Urulica | -21.5944 | -65.8322 | Tupiza | 1 | Highlands |
| CV-136(258) cl5 | 1.04.10 | *Triatoma infestans* | Tupiza/Urulica | -21.5944 | -65.8322 | Tupiza | 1 | Highlands |
| CV-137(259) cl4^*^ | 1.04.10 | *Triatoma infestans* | Tupiza/Palquiza | -21.5292 | -65.7513 | Tupiza | 8 | Highlands |
| CV-137(259) cl5 | 1.04.10 | *Triatoma infestans* | Tupiza/Palquiza | -21.5292 | -65.7513 | Tupiza | 10 | Highlands |
| CV-139(260) cl1 | 1.04.10 | *Triatoma infestans* | Tupiza/Palquiza | -21.5292 | -65.7513 | Tupiza | 1 | Highlands |
| CV-139(260) cl2^*^ | 1.04.10 | *Triatoma infestans* | Tupiza/Palquiza | -21.5292 | -65.7513 | Tupiza | 8 | Highlands |
| CV-139(260) cl3 | 1.04.10 | *Triatoma infestans* | Tupiza/Palquiza | -21.5292 | -65.7513 | Tupiza | 10 | Highlands |
| CV-142(261) cl1^*^ | 17.05.10 | *Triatoma infestans* | Toro Toro/Julo Chico | -18.012 | -65.8097 | Toro Toro | 1 | Highlands |
| CV-142(261) cl2^*^ | 17.05.10 | *Triatoma infestans* | Toro Toro/Julo Chico | -18.012 | -65.8097 | Toro Toro | 1 | Highlands |
| CV-143(262) cl3^*^ | 17.05.10 | *Triatoma infestans* | Toro Toro/Julo Chico | -18.012 | -65.8097 | Toro Toro | 8 | Highlands |
| CV-143(262) cl9^*^ | 17.05.10 | *Triatoma infestans* | Toro Toro/Julo Chico | -18.012 | -65.8097 | Toro Toro | 1 | Highlands |
| CV-144(280) cl2^*^ | 17.05.10 | *Triatoma infestans* | Toro Toro/Julo Chico | -18.0121 | -65.8096 | Toro Toro | 1 | Highlands |
| CV-144(280) cl4^*^ | 17.05.10 | *Triatoma infestans* | Toro Toro/Julo Chico | -18.0121 | -65.8096 | Toro Toro | 1 | Highlands |
| CV-145(263) cl1 | 17.05.10 | *Triatoma infestans* | Toro Toro/Julo Chico | -18.0121 | -65.8096 | Toro Toro | 1 | Highlands |
| CV-145(263) cl2^*^ | 17.05.10 | *Triatoma infestans* | Toro Toro/Julo Chico | -18.0121 | -65.8096 | Toro Toro | 1 | Highlands |
| CV-145(263) cl3 | 17.05.10 | *Triatoma infestans* | Toro Toro/Julo Chico | -18.0121 | -65.8096 | Toro Toro | 1 | Highlands |
| CV-147(265) cl1^*^ | 17.05.10 | *Triatoma infestans* | Toro Toro/Julo Chico | -18.0123 | -65.8088 | Toro Toro | 8 | Highlands |
| CV-147(265) cl2 | 17.05.10 | *Triatoma infestans* | Toro Toro/Julo Chico | -18.0123 | -65.8088 | Toro Toro | 8 | Highlands |
| CV-147(265) cl3 | 17.05.10 | *Triatoma infestans* | Toro Toro/Julo Chico | -18.0123 | -65.8088 | Toro Toro | 8 | Highlands |
| CV-148(273) cl3^*^ | 17.05.10 | *Triatoma infestans* | Toro Toro/Julo Chico | -18.0122 | -65.8091 | Toro Toro | 8 | Highlands |
| CV-148(273) cl4 | 17.05.10 | *Triatoma infestans* | Toro Toro/Julo Chico | -18.0122 | -65.8091 | Toro Toro | 10 | Highlands |
| CV-148(273) cl5 | 17.05.10 | *Triatoma infestans* | Toro Toro/Julo Chico | -18.0122 | -65.8091 | Toro Toro | 10 | Highlands |
| CV-149(305) cl2 | 17.05.10 | *Triatoma infestans* | Toro Toro/Julo Chico | -18.0122 | -65.8091 | Toro Toro | 8 | Highlands |
| CV-149(305) cl3^*^ | 17.05.10 | *Triatoma infestans* | Toro Toro/Julo Chico | -18.0122 | -65.8091 | Toro Toro | 8 | Highlands |
| CV-180(238) cl4^*^ | 21.05.10 | *Triatoma infestans* | Campero/Huertas | -18.2441 | -64.8615 | Toro Toro | 8 | Highlands |
| CV-180(238) cl5^*^ | 21.05.10 | *Triatoma infestans* | Campero/Huertas | -18.2441 | -64.8615 | Toro Toro | 8 | Highlands |
| CV-181(274) cl1^*^ | 21.05.10 | *Triatoma infestans* | Campero/Huertas | -18.2441 | -64.8615 | Toro Toro | 8 | Highlands |
| CV-181(274) cl2^*^ | 21.05.10 | *Triatoma infestans* | Campero/Huertas | -18.2441 | -64.8615 | Toro Toro | 8 | Highlands |
| CV-225(276) cl1 | 26.05.10 | *Triatoma guasayana* | Campero/Camino Mesada | -18.195 | -64.8635 | Toro Toro | 10 | Highlands |
| CV-225(276) cl5^*^ | 26.05.10 | *Triatoma guasayana* | Campero/Camino Mesada | -18.195 | -64.8635 | Toro Toro | 8 | Highlands |
| CV-245(309) cl1 | 17.05.10 | *Triatoma infestans* | Toro Toro/Julo Grande | -18.016 | -65.8 | Toro Toro | 10 | Highlands |
| CV-245(309) cl2 | 17.05.10 | *Triatoma infestans* | Toro Toro/Julo Grande | -18.016 | -65.8 | Toro Toro | 8 | Highlands |
| CV-245(309) cl3^*^ | 17.05.10 | *Triatoma infestans* | Toro Toro/Julo Grande | -18.016 | -65.8 | Toro Toro | 8 | Highlands |
| CV-249 cl3^*^ | 17.05.10 | *Triatoma infestans* | Toro Toro/Julo Grande | -18.0123 | -65.8083 | Toro Toro | 8 | Highlands |
| CV-249 cl6 | 17.05.10 | *Triatoma infestans* | Toro Toro/Julo Grande | -18.0123 | -65.8083 | Toro Toro | 8 | Highlands |
| CV-254(442) cl5^*^ | 17.05.10 | *Triatoma infestans* | Toro Toro/Julo Grande | -18.0123 | -65.8083 | Toro Toro | 8 | Highlands |
| CV-254(442) cl6^*^ | 17.05.10 | *Triatoma infestans* | Toro Toro/Julo Grande | -18.0123 | -65.8083 | Toro Toro | 1 | Highlands |
| CV-255(507) cl5^*^ | 17.05.10 | *Triatoma infestans* | Toro Toro/Julo Grande | -18.0123 | -65.8083 | Toro Toro | 10 | Highlands |
| CV-255(507) cl6^*^ | 17.05.10 | *Triatoma infestans* | Toro Toro/Julo Grande | -18.0123 | -65.8083 | Toro Toro | 8 | Highlands |
| CV-256 cl1 | 17.05.10 | *Triatoma infestans* | Toro Toro/Julo Grande | -18.0123 | -65.8083 | Toro Toro | 8 | Highlands |
| CV-256 cl4^*^ | 17.05.10 | *Triatoma infestans* | Toro Toro/Julo Grande | -18.0123 | -65.8083 | Toro Toro | 8 | Highlands |
| CV-256 cl7 | 17.05.10 | *Triatoma infestans* | Toro Toro/Julo Grande | -18.0123 | -65.8083 | Toro Toro | 8 | Highlands |
| CV-257 cl1 | 17.05.10 | *Triatoma infestans* | Toro Toro/Julo Grande | -18.0123 | -65.8083 | Toro Toro | 8 | Highlands |
| CV-257 cl2^*^ | 17.05.10 | *Triatoma infestans* | Toro Toro/Julo Grande | -18.0123 | -65.8083 | Toro Toro | 10 | Highlands |
| CV-257 cl3 | 17.05.10 | *Triatoma infestans* | Toro Toro/Julo Grande | -18.0123 | -65.8083 | Toro Toro | 10 | Highlands |
| CV-258 cl2 | 17.05.10 | *Triatoma infestans* | Toro Toro/Julo Grande | -18.0123 | -65.8083 | Toro Toro | 8 | Highlands |
| CV-258 cl3 | 17.05.10 | *Triatoma infestans* | Toro Toro/Julo Grande | -18.0123 | -65.8083 | Toro Toro | 10 | Highlands |
| CV-258 cl5^*^ | 17.05.10 | *Triatoma infestans* | Toro Toro/Julo Grande | -18.0123 | -65.8083 | Toro Toro | 10 | Highlands |
| CV-059(236) cl1^*^ | 9.11.09 | *Triatoma infestans* | Campero/Ilicuni | -18.1589 | -64.8666 | Toro Toro | 10 | Highlands |
| CV-059(236) cl2^*^ | 9.11.09 | *Triatoma infestans* | Campero/Ilicuni | -18.1589 | -64.8666 | Toro Toro | 8 | Highlands |
| NUAL1 cl1 | 27.06.07 | *Rhodnius pictipes* | Nueva Alianza | -15.03 | -64.33 | East Beni | 7 | Lowlands 2 |
| NUAL1 cl2^*^ | 27.06.07 | *Rhodnius pictipes* | Nueva Alianza | -15.03 | -64.33 | East Beni | 7 | Lowlands 2 |
| NUAL1 cl3 | 27.06.07 | *Rhodnius pictipes* | Nueva Alianza | -15.03 | -64.33 | East Beni | 7 | Lowlands 2 |
| NUAL1 cl4 | 27.06.07 | *Rhodnius pictipes* | Nueva Alianza | -15.03 | -64.33 | East Beni | 7 | Lowlands 2 |
| SJM18 cl1 | 5.9.04 | *Didelphis marsupialis* | San Juan de Aguas Dulces, Beni | -14.81 | -64.6 | East Beni | 3 | Lowlands 1 |
| SJM18 cl2^*^ | 5.9.04 | *Didelphis marsupialis* | San Juan de Aguas Dulces, Beni | -14.81 | -64.6 | East Beni | 3 | Lowlands 1 |
| SJM18 cl3 | 5.9.04 | *Didelphis marsupialis* | San Juan de Aguas Dulces, Beni | -14.81 | -64.6 | East Beni | 2 | Lowlands 1 |
| SJM18 cl4 | 5.9.04 | *Didelphis marsupialis* | San Juan de Aguas Dulces, Beni | -14.81 | -64.6 | East Beni | 3 | Lowlands 1 |
| SJM22 cl1 | 6.9.04 | *Didelphis marsupialis* | San Juan de Aguas Dulces, Beni | -14.81 | -64.6 | East Beni | 6 | Lowlands 1 |
| SJM22 cl2^*^ | 6.9.04 | *Didelphis marsupialis* | San Juan de Aguas Dulces, Beni | -14.81 | -64.6 | East Beni | 6 | Lowlands 1 |
| SJM22 cl4 | 6.9.04 | *Didelphis marsupialis* | San Juan de Aguas Dulces, Beni | -14.81 | -64.6 | East Beni | 6 | Lowlands 1 |
| SJM23 cl1 | 6.9.04 | *Didelphis marsupialis* | San Juan de Aguas Dulces, Beni | -14.81 | -64.6 | East Beni | 6 | Lowlands 1 |
| SJM23 cl4 | 6.9.04 | *Didelphis marsupialis* | San Juan de Aguas Dulces, Beni | -14.81 | -64.6 | East Beni | 6 | Lowlands 1 |
| SJM23 cl5 | 6.9.04 | *Didelphis marsupialis* | San Juan de Aguas Dulces, Beni | -14.81 | -64.6 | East Beni | 6 | Lowlands 1 |
| SJM23 cl6^*^ | 6.9.04 | *Didelphis marsupialis* | San Juan de Aguas Dulces, Beni | -14.81 | -64.6 | East Beni | 6 | Lowlands 1 |
| SJM25 cl1^*^ | 6.9.04 | *Didelphis marsupialis* | San Juan de Aguas Dulces, Beni | -14.81 | -64.6 | East Beni | 7 | Lowlands 2 |
| SJM25 cl2 | 6.9.04 | *Didelphis marsupialis* | San Juan de Aguas Dulces, Beni | -14.81 | -64.6 | East Beni | 7 | Lowlands 2 |
| SJM25 cl3 | 6.9.04 | *Didelphis marsupialis* | San Juan de Aguas Dulces, Beni | -14.81 | -64.6 | East Beni | 7 | Lowlands 2 |
| SJM25 cl4 | 6.9.04 | *Didelphis marsupialis* | San Juan de Aguas Dulces, Beni | -14.81 | -64.6 | East Beni | 7 | Lowlands 2 |
| SJM26 cl1^*^ | 6.9.04 | *Didelphis marsupialis* | San Juan de Aguas Dulces, Beni | -14.81 | -64.6 | East Beni | 6 | Lowlands 1 |
| SJM26 cl2 | 6.9.04 | *Didelphis marsupialis* | San Juan de Aguas Dulces, Beni | -14.81 | -64.6 | East Beni | 6 | Lowlands 1 |
| SJM32 cl1 | 7.9.04 | *Philander opossum* | San Juan de Aguas Dulces, Beni | -14.81 | -64.6 | East Beni | 9 | Lowlands 2 |
| SJM32 cl2 | 7.9.04 | *Philander opossum* | San Juan de Aguas Dulces, Beni | -14.81 | -64.6 | East Beni | 9 | Lowlands 2 |
| SJM32 cl3^*^ | 7.9.04 | *Philander opossum* | San Juan de Aguas Dulces, Beni | -14.81 | -64.6 | East Beni | 9 | Lowlands 2 |
| SJM32 cl4 | 7.9.04 | *Philander opossum* | San Juan de Aguas Dulces, Beni | -14.81 | -64.6 | East Beni | 9 | Lowlands 2 |
| SJM33 cl1 | 7.9.04 | *Didelphis marsupialis* | San Juan de Aguas Dulces, Beni | -14.81 | -64.6 | East Beni | 6 | Lowlands 1 |
| SJM33 cl4 | 7.9.04 | *Didelphis marsupialis* | San Juan de Aguas Dulces, Beni | -14.81 | -64.6 | East Beni | 6 | Lowlands 1 |
| SJM34 cl1^*^ | 7.9.04 | *Didelphis marsupialis* | San Juan de Aguas Dulces, Beni | -14.81 | -64.6 | East Beni | 2 | Lowlands 1 |
| SJM34 cl4 | 7.9.04 | *Didelphis marsupialis* | San Juan de Aguas Dulces, Beni | -14.81 | -64.6 | East Beni | 2 | Lowlands 1 |
| SJM35 cl1 | 7.9.04 | *Didelphis marsupialis* | San Juan de Aguas Dulces, Beni | -14.81 | -64.6 | East Beni | 7 | Lowlands 2 |
| SJM35 cl2^*^ | 7.9.04 | *Didelphis marsupialis* | San Juan de Aguas Dulces, Beni | -14.81 | -64.6 | East Beni | 7 | Lowlands 2 |
| SJM35 cl3 | 7.9.04 | *Didelphis marsupialis* | San Juan de Aguas Dulces, Beni | -14.81 | -64.6 | East Beni | 7 | Lowlands 2 |
| SJM37 cl1^*^ | 9.9.04 | *Didelphis marsupialis* | San Juan de Aguas Dulces, Beni | -14.81 | -64.6 | East Beni | 4 | Lowlands 2 |
| SJM37 cl2 | 9.9.04 | *Didelphis marsupialis* | San Juan de Aguas Dulces, Beni | -14.81 | -64.6 | East Beni | 4 | Lowlands 2 |
| SJM37 cl3 | 9.9.04 | *Didelphis marsupialis* | San Juan de Aguas Dulces, Beni | -14.81 | -64.6 | East Beni | 4 | Lowlands 2 |
| SJM37 cl4 | 9.9.04 | *Didelphis marsupialis* | San Juan de Aguas Dulces, Beni | -14.81 | -64.6 | East Beni | 4 | Lowlands 2 |
| SJM39 cl1 | 9.9.04 | *Didelphis marsupialis* | San Juan de Aguas Dulces, Beni | -14.81 | -64.6 | East Beni | 2 | Lowlands 1 |
| SJM39 cl2 | 9.9.04 | *Didelphis marsupialis* | San Juan de Aguas Dulces, Beni | -14.81 | -64.6 | East Beni | 2 | Lowlands 1 |
| SJM39 cl3^*^ | 9.9.04 | *Didelphis marsupialis* | San Juan de Aguas Dulces, Beni | -14.81 | -64.6 | East Beni | 2 | Lowlands 1 |
| SJM39 cl4 | 9.9.04 | *Didelphis marsupialis* | San Juan de Aguas Dulces, Beni | -14.81 | -64.6 | East Beni | 2 | Lowlands 1 |
| SJM3 cl1 | 2.9.04 | *Didelphis marsupialis* | San Juan de Aguas Dulces, Beni | -14.81 | -64.6 | East Beni | 7 | Lowlands 2 |
| SJM3 cl2 | 2.9.04 | *Didelphis marsupialis* | San Juan de Aguas Dulces, Beni | -14.81 | -64.6 | East Beni | 7 | Lowlands 2 |
| SJM3 cl3 | 2.9.04 | *Didelphis marsupialis* | San Juan de Aguas Dulces, Beni | -14.81 | -64.6 | East Beni | 7 | Lowlands 2 |
| SJM3 cl4^*^ | 2.9.04 | *Didelphis marsupialis* | San Juan de Aguas Dulces, Beni | -14.81 | -64.6 | East Beni | 7 | Lowlands 2 |
| SJM40 cl1^*^ | 9.9.04 | *Didelphis marsupialis* | San Juan de Aguas Dulces, Beni | -14.81 | -64.6 | East Beni | 9 | Lowlands 2 |
| SJM40 cl2 | 9.9.04 | *Didelphis marsupialis* | San Juan de Aguas Dulces, Beni | -14.81 | -64.6 | East Beni | 9 | Lowlands 2 |
| SJM41 cl1 | 9.9.04 | *Philander opossum* | San Juan de Aguas Dulces, Beni | -14.81 | -64.6 | East Beni | 7 | Lowlands 2 |
| SJM41 cl2 | 9.9.04 | *Philander opossum* | San Juan de Aguas Dulces, Beni | -14.81 | -64.6 | East Beni | 7 | Lowlands 2 |
| SJM41 cl3^*^ | 9.9.04 | *Philander opossum* | San Juan de Aguas Dulces, Beni | -14.81 | -64.6 | East Beni | 7 | Lowlands 2 |
| SJMC3 cl1^*^ | 6.9.04 | *Didelphis marsupialis* | San Juan de Aguas Dulces, Beni | -14.81 | -64.6 | East Beni | 6 | Lowlands 1 |
| SJMC3 cl3 | 6.9.04 | *Didelphis marsupialis* | San Juan de Aguas Dulces, Beni | -14.81 | -64.6 | East Beni | 6 | Lowlands 1 |
| SJMC3 cl4 | 6.9.04 | *Didelphis marsupialis* | San Juan de Aguas Dulces, Beni | -14.81 | -64.6 | East Beni | 6 | Lowlands 1 |
| SJMC7 cl1 | 11.9.04 | *Scuireus sp.* | San Juan de Aguas Dulces, Beni | -14.81 | -64.6 | East Beni | 4 | Lowlands 2 |
| SJMC7 cl4^*^ | 11.9.04 | *Scuireus sp.* | San Juan de Aguas Dulces, Beni | -14.81 | -64.6 | East Beni | 4 | Lowlands 2 |
| SJMC7 cl6 | 11.9.04 | *Scuireus sp.* | San Juan de Aguas Dulces, Beni | -14.81 | -64.6 | East Beni | 4 | Lowlands 2 |
| SJMO11 cl1 | 21.06.07 | *Didelphis marsupialis* | San Juan de Mocovi | -15.116 | -64.316 | East Beni | 2 | Lowlands 1 |
| SJMO11 cl2 | 21.06.07 | *Didelphis marsupialis* | San Juan de Mocovi | -15.116 | -64.316 | East Beni | 2 | Lowlands 1 |
| SJMO11 cl3 | 21.06.07 | *Didelphis marsupialis* | San Juan de Mocovi | -15.116 | -64.316 | East Beni | 2 | Lowlands 1 |
| SJMO11 cl4^*^ | 21.06.07 | *Didelphis marsupialis* | San Juan de Mocovi | -15.116 | -64.316 | East Beni | 2 | Lowlands 1 |
| SJMO3 cl1 | 20.06.07 | *Didelphis marsupialis* | San Juan de Mocovi | -15.116 | -64.316 | East Beni | 6 | Lowlands 2 |
| SJMO3 cl2^*^ | 20.06.07 | *Didelphis marsupialis* | San Juan de Mocovi | -15.116 | -64.316 | East Beni | 6 | Lowlands 2 |
| SJMO3 cl3 | 20.06.07 | *Didelphis marsupialis* | San Juan de Mocovi | -15.116 | -64.316 | East Beni | 6 | Lowlands 2 |
| SJMO9 cl1 | 21.06.07 | *Didelphis marsupialis* | San Juan de Mocovi | -15.116 | -64.316 | East Beni | 2 | Lowlands 1 |
| SJMO9 cl3^*^ | 21.06.07 | *Didelphis marsupialis* | San Juan de Mocovi | -15.116 | -64.316 | East Beni | 2 | Lowlands 1 |
| SJMOR20 cl1 | 22.06.07 | *Rhodnius pictipes* | San Juan de Mocovi | -15.116 | -64.316 | East Beni | 5 | Lowlands 2 |
| SJMOR20 cl2 | 22.06.07 | *Rhodnius pictipes* | San Juan de Mocovi | -15.116 | -64.316 | East Beni | 5 | Lowlands 2 |
| SJMOR20 cl3^*^ | 22.06.07 | *Rhodnius pictipes* | San Juan de Mocovi | -15.116 | -64.316 | East Beni | 5 | Lowlands 2 |
| SJMOR20 cl4 | 22.06.07 | *Rhodnius pictipes* | San Juan de Mocovi | -15.116 | -64.316 | East Beni | 5 | Lowlands 2 |
| SJMOR21 cl1^*^ | 22.06.07 | *Rhodnius pictipes* | San Juan de Mocovi | -15.116 | -64.316 | East Beni | 3 | Lowlands 1 |
| SJMOR21 cl2 | 22.06.07 | *Rhodnius pictipes* | San Juan de Mocovi | -15.116 | -64.316 | East Beni | 3 | Lowlands 1 |
| SJMOR21 cl3 | 22.06.07 | *Rhodnius pictipes* | San Juan de Mocovi | -15.116 | -64.316 | East Beni | 3 | Lowlands 1 |
| SJMOR21 cl4 | 22.06.07 | *Rhodnius pictipes* | San Juan de Mocovi | -15.116 | -64.316 | East Beni | 3 | Lowlands 1 |
| SJMOR22 cl1 | 22.06.07 | *Rhodnius pictipes* | San Juan de Mocovi | -15.116 | -64.316 | East Beni | 5 | Lowlands 2 |
| SJMOR22 cl2 | 22.06.07 | *Rhodnius pictipes* | San Juan de Mocovi | -15.116 | -64.316 | East Beni | 5 | Lowlands 2 |
| SJMOR22 cl3^*^ | 22.06.07 | *Rhodnius pictipes* | San Juan de Mocovi | -15.116 | -64.316 | East Beni | 5 | Lowlands 2 |
| SJMOR22 cl4 | 22.06.07 | *Rhodnius pictipes* | San Juan de Mocovi | -15.116 | -64.316 | East Beni | 5 | Lowlands 2 |
| SJMOR23 cl2 | 22.06.07 | *Rhodnius pictipes* | San Juan de Mocovi | -15.116 | -64.316 | East Beni | 5 | Lowlands 2 |
| SJMOR23 cl3 | 22.06.07 | *Rhodnius pictipes* | San Juan de Mocovi | -15.116 | -64.316 | East Beni | 5 | Lowlands 2 |
| SJMOR23 cl4^*^ | 22.06.07 | *Rhodnius pictipes* | San Juan de Mocovi | -15.116 | -64.316 | East Beni | 5 | Lowlands 2 |
| SJMOR29 cl1 | 22.06.07 | *Rhodnius* | San Juan de Mocovi | -15.116 | -64.316 | East Beni | 9 | Lowlands 2 |
| SJMOR29 cl2 | 22.06.07 | *Rhodnius* | San Juan de Mocovi | -15.116 | -64.316 | East Beni | 9 | Lowlands 2 |
| SJMOR29 cl4 | 22.06.07 | *Rhodnius* | San Juan de Mocovi | -15.116 | -64.316 | East Beni | 9 | Lowlands 2 |
| SJMOR29 cl5^*^ | 22.06.07 | *Rhodnius* | San Juan de Mocovi | -15.116 | -64.316 | East Beni | 9 | Lowlands 2 |
| SJMOR30 cl1 | 22.06.07 | *Rhodnius* | San Juan de Mocovi | -15.116 | -64.316 | East Beni | 3 | Lowlands 1 |
| SJMOR30 cl2^*^ | 22.06.07 | *Rhodnius* | San Juan de Mocovi | -15.116 | -64.316 | East Beni | 3 | Lowlands 1 |
| SJMOR30 cl3 | 22.06.07 | *Rhodnius* | San Juan de Mocovi | -15.116 | -64.316 | East Beni | 3 | Lowlands 1 |
| SJMOR30 cl5 | 22.06.07 | *Rhodnius* | San Juan de Mocovi | -15.116 | -64.316 | East Beni | 3 | Lowlands 1 |
| MERC10 cl1^*^ | 15.06.07 | *Rhodnius robustus* | Mercedes | -14.73 | -65.73 | North Beni | 2 | Lowlands 1 |
| MERC10 cl2 | 15.06.07 | *Rhodnius robustus* | Mercedes | -14.73 | -65.73 | North Beni | 2 | Lowlands 1 |
| MERC10 cl3 | 15.06.07 | *Rhodnius robustus* | Mercedes | -14.73 | -65.73 | North Beni | 2 | Lowlands 1 |
| MERC10 cl4 | 15.06.07 | *Rhodnius robustus* | Mercedes | -14.73 | -65.73 | North Beni | 2 | Lowlands 1 |
| SCRIR6 cl1 | 23.07.07 | *Philander opossum* | San Cristobal | -14.13 | -66.92 | North Beni | 2 | Lowlands 1 |
| SCRIR6 cl2 | 23.07.07 | *Philander opossum* | San Cristobal | -14.13 | -66.92 | North Beni | 2 | Lowlands 1 |
| SCRIR6 cl3^*^ | 23.07.07 | *Philander opossum* | San Cristobal | -14.13 | -66.92 | North Beni | 2 | Lowlands 1 |
| SCRIR6 cl4 | 23.07.07 | *Philander opossum* | San Cristobal | -14.13 | -66.92 | North Beni | 2 | Lowlands 1 |
| SMA2 cl1 | 5.08.04 | *Didelphis marsupialis* | Santa Maria de Apere | -14.13 | -65.36 | North Beni | 2 | Lowlands 1 |
| SMA2 cl2 | 5.08.04 | *Didelphis marsupialis* | Santa Maria de Apere | -14.13 | -65.36 | North Beni | 2 | Lowlands 1 |
| SMA2 cl3^*^ | 5.08.04 | *Didelphis marsupialis* | Santa Maria de Apere | -14.13 | -65.36 | North Beni | 2 | Lowlands 1 |
| SMA2 cl4 | 5.08.04 | *Didelphis marsupialis* | Santa Maria de Apere | -14.13 | -65.36 | North Beni | 2 | Lowlands 1 |
| SMA4 cl1 | 5.08.04 | *Didelphis marsupialis* | Santa Maria de Apere | -14.13 | -65.36 | North Beni | 2 | Lowlands 1 |
| SMA4 cl2 | 5.08.04 | *Didelphis marsupialis* | Santa Maria de Apere | -14.13 | -65.36 | North Beni | 2 | Lowlands 1 |
| SMA4 cl3^*^ | 5.08.04 | *Didelphis marsupialis* | Santa Maria de Apere | -14.13 | -65.36 | North Beni | 2 | Lowlands 1 |
| SMA5 cl1 | 5.08.04 | *Didelphis marsupialis* | Santa Maria de Apere | -14.13 | -65.36 | North Beni | 2 | Lowlands 1 |
| SMA5 cl2^*^ | 5.08.04 | *Didelphis marsupialis* | Santa Maria de Apere | -14.13 | -65.36 | North Beni | 2 | Lowlands 1 |
| SMA5 cl4 | 5.08.04 | *Didelphis marsupialis* | Santa Maria de Apere | -14.13 | -65.36 | North Beni | 2 | Lowlands 1 |
| SMA5 cl5 | 5.08.04 | *Didelphis marsupialis* | Santa Maria de Apere | -14.13 | -65.36 | North Beni | 2 | Lowlands 1 |
| SMA6 cl1^*^ | 5.08.04 | *Didelphis marsupialis* | Santa Maria de Apere | -14.13 | -65.36 | North Beni | 2 | Lowlands 1 |
| SMA6 cl3 | 5.08.04 | *Didelphis marsupialis* | Santa Maria de Apere | -14.13 | -65.36 | North Beni | 2 | Lowlands 1 |
| SMA6 cl4 | 5.08.04 | *Didelphis marsupialis* | Santa Maria de Apere | -14.13 | -65.36 | North Beni | 2 | Lowlands 1 |
| SMR37 cl1 | 5.09.04 | *Rhodnius* | Santa Maria de Apere | -14.13 | -65.36 | North Beni | 2 | Lowlands 1 |
| SMR37 cl2^*^ | 5.09.04 | *Rhodnius* | Santa Maria de Apere | -14.13 | -65.36 | North Beni | 2 | Lowlands 1 |
| SMR37 cl3 | 5.09.04 | *Rhodnius* | Santa Maria de Apere | -14.13 | -65.36 | North Beni | 2 | Lowlands 1 |
| SMR37 cl4 | 5.09.04 | *Rhodnius* | Santa Maria de Apere | -14.13 | -65.36 | North Beni | 2 | Lowlands 1 |

^a^ Latitude and longitude in decimal degrees.

^b^ *A priori* population assignment based on geographical origin: Cochabamba, Tupiza, Toro Toro, North Beni and East Beni (Figure 1).

^c^ *A posteriori* genetic population assignment via discriminate analysis of principal components (DAPC) (Jombart *et al.* 2010) (Figure 2).

^d^ Genetic population assignment based on position in the *D*_AS_ topology (Figure 3).

* Indicates clones for which ten maxicircle gene fragments were sequence and concatenated according to Messenger *et al.* 2012.
